# Histopathological patterns and immunophenotyping of feline lymphomas and incidence in Metropolitan Bangkok, Thailand

**DOI:** 10.14202/vetworld.2024.2225-2234

**Published:** 2024-10-04

**Authors:** Jedsada Siripoonsub, Sirintra Sirivisoot, Somporn Techangamsuwan, Anudep Rungsipipat

**Affiliations:** Center of Excellence for Companion Animal Cancer, Department of Pathology, Faculty of Veterinary Science, Chulalongkorn University, Bangkok, 10330 Thailand

**Keywords:** B-cell, feline, immunophenotype, incidence, lymphoma, t-cell

## Abstract

**Background and Aim:**

Feline lymphomas are categorized based on the location of tumor cells, with anatomical classifications including alimentary, mediastinal, multicentric, and extranodal forms. Accurate diagnosis and classification of feline lymphoma are paramount for enhancing treatment and prognosis. T-cell lymphomas are CD3 positive, while B-cell lymphomas exhibit positive forCD20, CD79α, and paired box 5 (PAX5). The aims of this study were (1) to classify feline lymphoma in each anatomical subtype using the World Health Organization (WHO) classification to provide information on epidemiological findings; (2) to investigate the expression and detection of B-cell lymphoma, various antibodies will be used, with the addition of PAX5, for clearer results; and (3) to gather more extensive information about feline lymphoma in Thailand, particularly in the Bangkok area.

**Materials and Methods:**

From 2011 to 2023, 86 sample tissues were submitted for routine pathological examination at the Department of Pathology, Faculty of Veterinary Science, Chulalongkorn University. Immunohistochemistry (IHC) was performed to detect an immunophenotype of PAX5, CD79α, CD20 (B-cell lineage), and CD3 (T-cell lineage). Eighty-six formalin-fixed, paraffin-embedded lymphoma tissues were prepared on silane-coated slides. After IHC, all cases were classified according to the WHO classification.

**Results:**

The most common form of lymphoma in this study was extranodal lymphoma at 37.2% (32/86), followed by multicentric lymphoma at 31.3% (27/74), mediastinal lymphoma at 17.4% (15/86), and alimentary lymphoma at 14% (12/86). Most extranodal lymphoma cases were in the nasal region. From the anatomical form, multicentric and extranodal lymphomas were predominantly diffuse large B-cell high-grade, while mediastinal lymphomas were small low-grade B-cell lymphomas. Alimentary lymphomas occur in various types, with most being the B-cell type.

**Conclusion:**

This study indicates that extranodal lymphoma and extranodal lymphoma are the most frequent presentations found in cats in Bangkok. Mediastinal and alimentary lymphomas still occur. The utilization of various B-cell markers in combination could aid pathologists in distinguishing between various stages of B-cell maturation, assessing tumor cell heterogeneity, and determining the phenotype in scenarios where there is a loss of common B-cell markers diffuse large B-cell lymphomas is the most prevalent subtype of feline lymphoma. Significantly, relying solely on immunochemistry with one parameter may not be sufficient for a definitive diagnosis of B-cell lymphoma, as another parameter may also be necessary.

## Introduction

Feline lymphoma is a type of cancer affecting the hematopoietic system and is the most frequently diagnosed neoplasm in domestic cats. Hematopoietic tumors are estimated to make up about one-third of all feline tumors, with lymphoma representing 50%–90% of these cases [1, 2]. This condition accounts for a significant one-third of all tumors observed in cats. Lymphoma is a type of cancer characterized by the uncontrolled proliferation of malignant lymphoid cells, originating from tissues outside the bone marrow [3]. These cancers are categorized based on the location of tumor cells, with anatomical classifications encompassing alimentary, mediastinal, multicentric, and extranodal forms [4, 5]. The most common anatomical site is the alimentary form compared with other sites [2, 6–8]. Extranodal lymphoma is more frequently observed in cats than in multicentric form [9]. Extranodal presentation is currently the most frequent form of lymphoma in cats, often involving the abdomen and occurring in atypical locations [10]. A previous study by Sato *et al*. [11] observed that the anatomical location of lymphoma in cats influenced the median survival time. Specifically, cats with alimentary lymphoma exhibited a significantly shorter median survival than those with nasal or mediastinal lymphoma.

A definitive diagnosis is made using a combination of tools, including cytology, histopathology, immunophenotyping, and molecular methods [12]. Histopathology is considered the gold standard for diagnosing lymphoma [13], while immunophenotyping is a useful diagnostic tool for lymphoid neoplasms and can assist with histopathological classification [14]. Moreover, it is a straightforward and time-efficient technique for assessing protein expression in tissues, making it a valuable tool for cancer research [14]. However, inadequate sample size, the presence of non-neoplastic cells, reactive hyperplastic lesions, or early stages of lymphoma can affect the accuracy of diagnosis, which can influence the treatment and prognosis. Accurate diagnosis and classification are crucial for improving treatment and prognosis. T-cell lymphomas are CD3 positive, while B-cell lymphomas are positive for CD20, CD79α, and paired box 5 (PAX5). In some cases, CD20 and CD79α can lose immunoreactivity, which can occur after treatment with rituximab therapy [15]. PAX5 is a B-cell marker that is expressed throughout B-cell development and maturation but absent in plasma cells; its accuracy is similar to that of CD79α [14]. Various classification systems for non-Hodgkin’s lymphomas in humans have been adapted for use in the veterinary field. In 2002, the World Health Organization (WHO) published the Revised European-American Lymphoma classification for domestic animals. The WHO classification system can also be applied to feline lymphoma [1, 16]. The classification was adapted from human medicine and further enhanced by incorporating other hematopoietic myeloid tumors, guided by the expertise of the WHO panel of veterinary specialists [1]. In humans, the treatment protocol depends on the subtype of lymphoma. The WHO classification in veterinary medicine is similar to human medicine, though some subtypes differ [17]. The correlation between the lymphoma subtype with the anatomical site and treatment remains unknown [17]. Determining the immunologic phenotype and histologic classification of lymphomas in cats is crucial. B-cell lymphomas generally have a better response rate than T-cell lymphomas [18]. Previous studies by Ota-Kuroki *et al*. [18] have shown that animals with low-grade lymphoma, such as marginal zone lymphoma, follicular lymphoma, B-small lymphocytic lymphoma, T-cell-rich B-cell lymphoma, and T-zone lymphoma, can experience long-term survival, even without extensive chemotherapy. The data reporting on feline lymphoma, especially biphenotypic B-cell lymphoma, suggest the potential for the aggressive and unpredictable clinical behavior of this disease [19]. Precise immunophenotype characterization and classification are essential for achieving a more effective treatment approach and prognosis assessment. While numerous studies on lymphoma classification have been conducted in various countries, few studies in Thailand have explored this issue [2, 8, 20].

The aims of this study were (1) to classify feline lymphoma in each anatomical subtype using the WHO classification to provide information on epidemiological findings; (2) to investigate the expression and detection of B-cell lymphoma, various antibodies will be used, with the addition of PAX5, for more precise results; and (3) to gather more extensive information about feline lymphoma in Thailand, particularly in the Bangkok area.

## Materials and Methods

### Ethical approval

The study was approved by a statement to confirm that all methods were carried out in accordance with relevant guidelines and regulations and sampling procedures were approved by the Chulalongkorn University Animal Care and Use Committee (No. 1831060). The study was conducted in accordance with the local legislation and institutional requirements.

### Study period and location

This retrospective study was conducted from August-2019 to May-2023 at the Department of Pathology, Faculty of Veterinary Science, Chulalongkorn University, Bangkok.

### Case selection

Tissue specimens were obtained from cases displaying generalized lymphadenopathy, retrobulbar or ocular masses, skin nodules, and nasal masses suspected of lymphoma. Several cases were identified through ultrasound or X-ray imaging, revealing enlargement of the intestine or abnormal masses. Subsequently, biopsy procedures were performed. Mediastinal forms were suspected by X-ray, pre- and post-thoracocentesis and fluid analysis was performed. Retrobulbar and nasal lymphoma cases underwent X-ray examinations to investigate masses and bone lysis. Following this, a cytology diagnosis was conducted, after which biopsies were performed. Some cases, particularly those with the mediastinal form, were collected post-treatment after their death. In addition, some cases were collected from the necropsy room following a sudden death, revealing masses suspected to be lymphoma, particularly in alimentary, mediastinal, or renal forms.

### Tissue preparation

Eighty-six cases of lymphoma were scrutinized, including extranodal lymphoma (n = 32), multicentric lymphoma (n = 27), mediastinal lymphoma (n = 15), and alimentary lymphoma (n = 12). All tissues were submitted for routine pathological examination at the Department of Pathology, Faculty of Veterinary Science, Chulalongkorn University, Bangkok, from 2011 to 2023. Clinical data were collected for all tissues, including ages, breeds, and clinical signs. Subsequently, the anatomical classification was based on the criteria outlined in Ettinger’s guidelines [7]. The lymph nodes or masses were fixed in 10% neutral buffered formalin, routinely histologically processed, and stained with hematoxylin and eosin. The section’s thickness was 4–6 μm. The criteria employed to identify types and subtypes are from the WHO’s monograph on hematopoietic tumors of domestic animals, considering the vast literature on the subject [1]. The criteria for distinguishing between low-grade and high-grade involved categorizing small lymphocytes (with a nuclear diameter <2 red blood cell diameters) as indicative of low-grade, while large lymphocytes (with a nuclear diameter >2 red blood cell diameters) suggested high-grade [21].

### Immunophenotyping and WHO classification

Immunohistochemistry (IHC) was performed to detect an immunophenotype of PAX5, CD79α CD20 (B-cell lineage), and CD3 (T-cell lineage). Eighty-six formalin-fixed paraffin-embedded lymphoma tissues were prepared on a silane-coated slide. For CD3 and CD79α, slides were treated with a 1% citrate buffer solution with a pH of 6.0 using a 95–100°C water bath for 20 min. CD20 skips the pre-treated step. For PAX5, slides were treated with a Tris ethylenediaminetetraacetic acid solution with a pH 9 and autoclaved at 121°C for 5 min. Endogenous peroxidase activity was quenched by incubating the sections for 30 min in 0.3% hydrogen peroxidase in phosphate-buffered saline and blocking them with 2.5% bovine serum albumin at 37°C for 30 min. The sections were incubated with the panel of primary antibodies specific to CD3 (T-cell marker, ready to use, Dako, Denmark), PAX5 (B-cell marker, dilution 1:50, Dako, Denmark), CD79α (B-cell marker, dilution 1:200, SCBT, USA), and CD20 (B-cell marker, dilution 1:300, Abcam, USA) for 90 min. Only PAX5 should be overnight at 4°C. Negative and positive control sections were also incubated at this stage. The positive control consisted of normal lymph nodes, while the negative control involved normal skin. Subsequently, they were incubated with Envision Polymer (Dako, Denmark), and 3,3’-diaminobenzidine was used as a chromogen. Slides were counterstained in Mayer’s hematoxylin, with the positive cytoplasmic staining of CD3, CD79α, and CD20. In contrast, the nuclear positive of PAX5 was evaluated as at least 60% of tumor-positive cells and diagnosed as T-cell or B-cell lymphomas (Table-1).

**Table-1:** Summary of antibodies for T-cell and B-cell lymphomas used in this study.

Primary antibodies	Dilution of primary antibodies	Commercial reference	Incubation condition	Secondary antibodies	Commercial reference
CD20	1:300	Abcam, MA, USA	1.30 h at 37°C	Envision	Dako, Glostrup, Denmark
CD79α	1:200	SCBT, Dallas, USA	1.30 h at 37°C	Envision	Dako, Glostrup, Denmark
PAX5	1:50	DAKO, CA, USA	4°C overnight	Envision	Dako, Glostrup, Denmark
CD3	Ready to use	DAKO, IR503, Denmark	1.30 h at 37°C	Envision	Dako, Glostrup, Denmark

The criteria for distinguishing between low-grade and high-grade involved categorizing small lymphocytes (with a nuclear diameter <2 red blood cell diameters) as indicative of low-grade, while large lymphocytes (with a nuclear diameter >2 red blood cell diameters) suggested high-grade [21]. Criteria for WHO classification were based on previous studies by Vezzali *et al*. [1], Ponce *et al*. [16], and Valli *et al*. [22].

## Results

The age of cats in this study ranged from 4 months to 3 years, with most cases occurring in cats aged 5 months to 3 years old. Phenotypes of 5 months to 3 years were B-cells. The proportion of female-to-male cases was 43 (50%) and 43 (50%), respectively. The domestic shorthair cat was the most observed breed in this study (Table-2). However, there were no records of vaccination status. In most cases, the status of feline leukemia virus (FeLV) and feline immunodeficiency virus (FIV) infections was not recorded. The most common form of lymphoma in this study was extranodal lymphoma at 37.2% (32/86), followed by multicentric lymphoma at 31.3% (27/74), mediastinal lymphoma at 17.4% (15/86), and alimentary lymphoma at 14% (12/86). Most extranodal lymphoma cases were in the nasal region. From the anatomical form, multicentric and extranodal lymphomas were predominantly diffuse large B-cell high-grade, while mediastinal lymphomas were small low-grade B-cell lymphomas. Alimentary lymphomas occur in various types, mostly the B-cell type (Tables-3 and 4).

**Table-2:** Characteristics of 86 feline lymphoma cases in this study.

Variables	Percentage (cases)
Breed	
Domestic short hair	95 (82/86)
Persian	4 (3/86)
Scottish fold	1 (1/86)
Sex	
Male	50 (43/86)
Female	50 (43/86)
Age (year)	
0.4–3	43 (37/86)
4–6	37 (32/86)
>6	20 (17/86)
Anatomical classification (86)	
Multicentric	31.3 (27/86)
Mediastinal	17.4 (15/86)
Alimentary	14 (12/86)
Extranodal	
Ocular	10.4 (9/86)
Renal	6 (5/86)
Nasal	11.6 (10/86)
Cutaneous	9.3 (8/86)
T-cell lymphoma	21 (18/86)
Low-grade	10.5 (9/86)
High-grade	10.5 (9/86)
B-cell lymphoma	79 (68/86)
Low-grade	38.4 (33/86)
High-grade	40.6 (35/86)

**Table-3:** Classifications of 86 feline lymphomas according to WHO classification.

B-cell lymphoma (79%, 68/86)	Percentage (case)	T-cell lymphoma (21%, 18/86)	Percentage (case)
B-small lymphocytic lymphoma	13.2 (9/68)	T-cell lymphoblastic lymphoma	39 (7/18)
Lymphoplasmacytic lymphoma	22 (15/68)		
Follicular lymphoma Grade II	13.2 (9/68)		
Follicular lymphoma Grade III	5.8 (4/68)	Peripheral T-cell lymphoma	50 (9/18)
Diffuse large B-cell	36.8 (25/68)	Cutaneous non-epitheliotropic lymphoma	11 (2/18)
Large-cell immunoblastic lymphoma	9 (6/68)		

WHO=World Health Organization

**Table-4:** Feline lymphoma distribution by anatomical site and WHO classification.

WHO classification	Anatomical classification			Extranodal lymphoma (n = 32)			
	
	Multicentric (n = 27) (%)	Mediastinal (n = 15) (%)	Alimentary (n = 12) (%)	Renal (n = 5) (%)	Nasal (n = 10) (%)	Ocular (n = 9) (%)	Skin (n = 8) (%)
B-cell lymphoma							
B-small lymphocytic lymphoma	14.8 (4/27)	13.33 (2/15)		6.25 (2/32)	3.1 (1/32)		
Lymphoplasmacytic lymphoma	7.4 (2/27)	20 (3/15)	25 (3/12)		15.6 (5/32)		6.25 (2/32)
Follicular lymphoma Grade II	14.8 (4/27)		8.33 (1/12)		3.1 (1/32)	6.25 (2/32)	3.1 (1/32)
Follicular lymphoma Grade III	7.4 (2/27)		8.33 (1/12)		3.1 (1/32)		
Diffuse large B-cell	26 (7/27)	20 (3/15)	25 (3/12)	9.4 (3/32)	6.25 (2/32)	15.6 (5/32)	6.25 (2/32)
Large-cell immunoblastic lymphoma	11.1 (3/27)		8.33 (1/12)			3.1 (1/32)	3.1 (1/32)
T-cell lymphoma							
T-cell lymphoblastic lymphoma	3.7 (1/27)	33.34 (5/15)				3.1 (1/32)	
Peripheral T-cell lymphoma	14.8 (4/27)	13.33 (2/15)	25 (3/12)				
Cutaneous non-epitheliotropic lymphoma							6.25 (2/32)

WHO=World Health Organization

B-cells were large and predominantly diffuse, and T-cells were peripheral lymphomas. In addition, 79% were categorized as B small lymphocytic lymphomas (13.2%), lymphoplasmacytic lymphomas (15%), follicular lymphoma Grade II (13.2%), follicular lymphoma Grade III (4%), diffuse large B-cell (36.8%), and large-cell immunoblastic lymphomas (LCIBL) (9%), respectively. Moreover, 21% of T-cell lymphomas are categorized as T-cell lymphoblastic lymphomas (TCLL) (39%), peripheral T-cell lymphomas (50%), and cutaneous T-cell lymphomas (11%), respectively (Tables-3 and 4).

**Figure-1:**
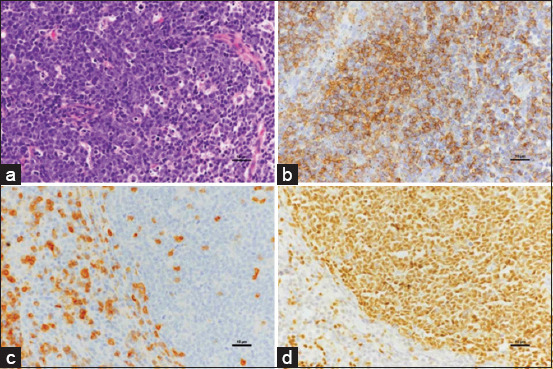
Lymph node, follicular lymphoma Grade II. (a) The tissue displays homogenous cell type and architecture, with few tingible body macrophages within the follicle (H&E). The nuclei of neoplastic cells were small to intermediate size. Moderate anisokaryosis is noted. The cytoplasm is minimal and highly basophilic. (b) The cytoplasm of neoplastic B-cells is densely positive for CD20 (IHC, hematoxylin counterstain). (c) Peripheral lymphoid tissue is scattered positive for CD3. (IHC, hematoxylin counterstain). (d) Strongly positive with a nuclear stain by PAX5 (IHC, hematoxylin counterstain). IHC=Immunohistochemistry, H&E=Hematoxylin and eosin.

Most lymphoma cases in this study were high-grade B cells. Multicentric lymphomas demonstrated a majority B-cell subtype, with diffuse large B-cell lymphomas (DLBCL). All cases depicted the clinical presence of more than one peripheral lymph node. Three cases presented with LCIBL. Follicular lymphoma cases primarily consisted of follicular center-cell lymphoma (Figures-1a–d). However, mantle-cell lymphoma and marginal-zone lymphoma were not detected in this study.

**Figure-2:**
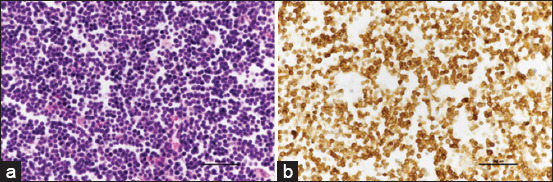
(a) Mediastinal mass diagnosed as T-cell lymphoblastic lymphoma is characterized by the infiltration of lymphoblastic cells, substantiated by a delicate fibrovascular stroma. The nuclei of these neoplastic cells display an intermediate to high size. Moderate anisokaryosis is observed. The cytoplasm is minimal and highly basophilic (H&E). (b) The cytoplasm of the neoplastic T-cells exhibits dense positive for CD3 (IHC, hematoxylin counterstain). IHC=Immunohistochemistry, H&E=Hematoxylin and eosin.

Mediastinal lymphomas demonstrated a relatively equal distribution between B-cell and T-cell subtypes, with TCLL representing the majority at 33% (5/15) (Figures-2a and b). According to the medical history, all cases of mediastinal lymphoma were diagnosed in domestic short-hair (DSH) cats and did not involve other lymph nodes. In addition, it was observed that all instances of TCLL occurred in cats aged between 5 months and 3 years. In cases of B-cell lymphomas, precursor cell neoplasms, and mature cell neoplasms were found.

**Figure-3:**
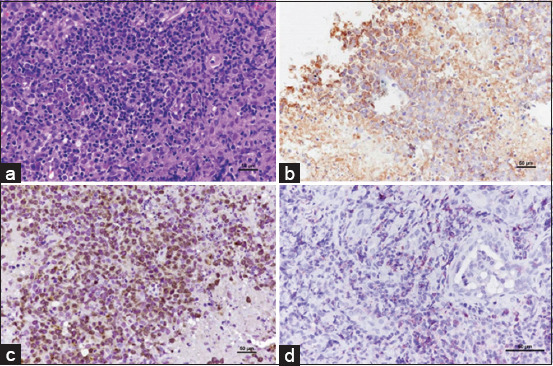
Nasal tissue diagnosed as lymphoplasmacytic lymphoma, lymphoblastic cell infiltration in the nasal mucosa and submucosa. The nuclei of neoplastic cells were small to intermediate size. Moderate anisokaryosis is noted. The cytoplasm is minimal and highly basophilic. The chromatin is densely stained and uniformly distributed, surrounding large, often central nucleolus (H&E). (b) Neoplastic cells are positive for CD20 (IHC, hematoxylin counterstain). (c) Positive for CD79α. Immunolabeling with anti-CD79α, (IHC, hematoxylin counterstain) (d) Multifocal Irregular cytoplasmic reactivity of small lymphocytes with CD3. Immunolabeling with anti-CD3 (IHC, hematoxylin counterstain). IHC=Immunohistochemistry, H&E=Hematoxylin and eosin.

**Figure-4:**
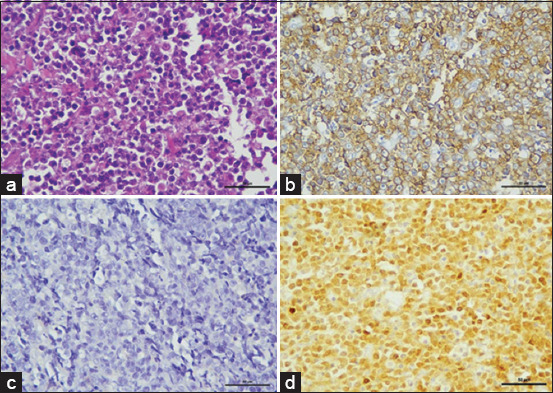
Retrobulbar mass diagnosed as diffuse large B-cell lymphoma, solid sheets of lymphoblastic cells supported by a fibrovascular stroma. The nuclei of neoplastic lymphoid cells are large (2 red cells in diameter) and have multiple prominent peripheral nucleoli. The chromatin is densely stained and uniformly distributed, surrounding large, often central nucleolus. There is abundant cytoplasm with indistinct cell boundaries (H&E). (b) Neoplastic cells are positive for CD20 (IHC, hematoxylin counterstain). (c) Negative with CD3 (IHC, hematoxylin counterstain). (d) Strongly positive with a nuclear stain by PAX5 (IHC, hematoxylin counterstain). IHC=Immunohistochemistry, H&E=Hematoxylin and eosin.

Extranodal lymphoma includes renal, nasal, ocular, and cutaneous forms. The predominant cases were nasal lymphomas, with the B-cell subtype being the most common. Among these, lymphoplasmacytic lymphoma emerged as the predominant subtype without epitheliotropism (Figures-3a–d). All cases showed predominantly male DSH. Ocular lymphomas were examined, encompassing intraocular and periocular locations, with most observed in periocular sites. The most prevalent subtype identified was DLBCL (Figures-4a–d).

**Figure-5:**
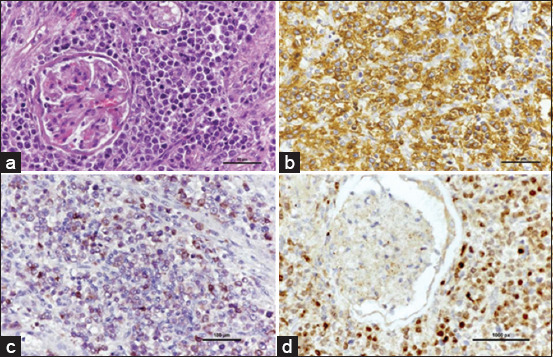
Kidney diagnosed as diffuse large B-cell lymphoma, lymphoblastic cells infiltration in renal parenchyma. The nuclei of neoplastic lymphoid cells are large (2 red cells in diameter) and have multiple prominent peripheral nucleoli. The chromatin is densely stained and uniformly distributed, surrounding large, often central nucleolus. There is abundant cytoplasm with indistinct cell boundaries (H&E) (b) Neoplastic cells are positive for CD20 (IHC, hematoxylin counterstain). (c) Multifocal Irregular cytoplasmic reactivity of small lymphocytes with CD3 (IHC, hematoxylin counterstain). (d) Strongly positive with a nuclear stain by PAX5 (IHC, hematoxylin counterstain). IHC=Immunohistochemistry, H&E=Hematoxylin and eosin.

Renal lymphoma primarily presents with B-cells, predominantly DLBCL (Figures-5a–d). In all cases, a solitary mass was observed in the renal area. A histological examination revealed the infiltration of lymphoblastic cells in the cortex, accompanied by renal tubular atrophy.

**Figure-6:**
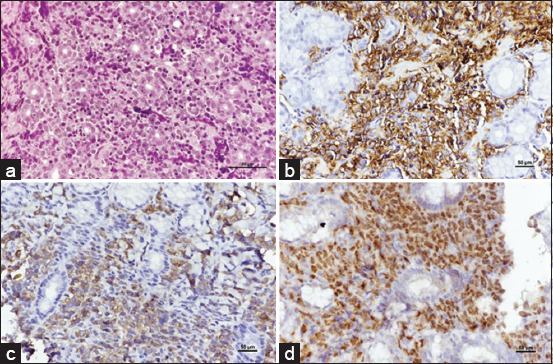
Alimentary lymphoma, stomach diagnosed as peripheral T-cell lymphoma. (a)The tissue displays homogenous cell type and architecture supported by delicate fibrovascular stroma at lamina propria and the submucosa (H&E). The nuclei of neoplastic cells were intermediate to large size. Moderate anisokaryosis is noted. The cytoplasm is minimal and highly basophilic. (b) The cytoplasm of neoplastic B-cells is densely positive for CD20 (IHC, hematoxylin counterstain). (c) Positive for CD79α. (IHC, hematoxylin counterstain). (d) Strongly positive with a nuclear stain by PAX5 (IHC, hematoxylin counterstain). IHC=Immunohistochemistry, H&E=Hematoxylin and eosin.

Alimentary lymphoma, which affects the entire gastrointestinal tract and mesenteric lymph nodes, presents a diverse range of lymphoma types, predominantly of B-cell origin (Figures-6a–d). Particularly in gastric locations, B-cell lymphoma was prevalent. Interestingly, out of 11 cases, 10 showed affected individuals over 4 years of age.

**Figure-7:**
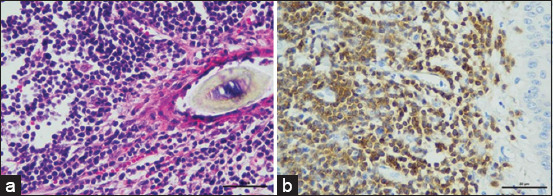
Skin, Cutaneous non-epitheliotropic lymphoma (a) Effacement of solid sheet architecture in the dermis. The nuclei of neoplastic lymphoid cells are large (2 red cells in diameter) and have a prominent peripheral nucleolus (H&E). (b) The neoplastic cells are positive for CD3 (IHC, hematoxylin counterstain). IHC=Immunohistochemistry, H&E=Hematoxylin and eosin.

Cutaneous lymphoma exhibited both B-cell and T-cell phenotypes. Specifically, two out of eight cases of cutaneous T-cell lymphoma showed no evidence of epitheliotropism (Figures-7a and b).

In some cases of B-cell lymphoma, CD79α may display a negative result, while PAX5 and CD20 may reveal a positive result. Importantly, in these cases, CD3 also typically depicts a negative result. No cross-reactivity was observed for PAX5 staining in CD3-positive T-cells (Table-5).

**Table-5:** Characteristics of feline lymphomas categorized by anatomical subtype, including comprehensive B-cell lineage markers (PAX5, CD20, and CD79α).

No.	Signalment				Immunophenotype		
	
	Breed	Sex	Age	Anatomical classification	PAX5	CD20	CD79α
1	DSH	M	4Y	Multicentric	+++	+++	+++
2	DSH	F	5Y	Multicentric	+++	+++	+
4	DSH	M	5Y	Multicentric	+++	+++	+++
10	DSH	F	6Y	Multicentric	+++	+++	+++
11	DSH	M	4Y	Multicentric	+	+++	+
13	DSH	F	4Y	Multicentric	+++	+++	+++
19	DSH	M	5M	Multicentric	+++	+++	+++
31	DSH	M	1Y	Multicentric	+++	+	+++
32	DSH	M	6Y	Multicentric	+++	+++	+++
63	DSH	F	5Y	Multicentric	+	+++	+
65	DSH	Fs	6Y	Multicentric	+++	+++	-
66	DSH	Fs	3Y	Multicentric	+++	+++	-
68	DSH	Mc	11Y	Multicentric	+++	+++	-
7	DSH	M	4Y	Mediastinal	+++	+++	-
48	DSH	F	8Y	Mediastinal	+	+++	+++
48	DSH	F	8Y	Mediastinal	+	+++	+++
52	DSH	F	2Y	Mediastinal	+++	+++	+++
60	DSH	M	1Y	Mediastinal	+++	+++	+++
67	DSH	Fs	8Y	Mediastinal	+++	+++	-
69	DSH	F	2Y	Mediastinal	+++	+++	-
51	DSH	F	6Y	Alimentary	+++	+++	+++
53	DSH	M	4Y	Alimentary	+++	+++	+
61	DSH	Fs	5Y	Alimentary	-	+++	+++
62	DSH	F	13Y	Alimentary	+++	+++	+++
73	DSH	Fs	14Y	Alimentary	+++	+++	+++
74	DSH	Mc	9Y	Alimentary	+++	+++	+++
17	Persia	F	7Y	Extranodal (renal)	+++	+++	+++
50	DSH	F	5Y	Extranodal (renal)	+++	+++	+++
70	DSH	F	6Y	Extranodal (renal)	+++	+++	+++
72	DSH	Fs	8Y	Extranodal (renal)	+++	+++	+++
18	DSH	M	5M	Extranodal (ocular)	+++	+++	+
20	DSH	M	4Y	Extranodal (ocular)	+++	+++	+++
37	DSH	M	5Y	Extranodal (ocular)	+++	+++	+
41	Persia	M	1.5y	Extranodal (skin)	+++	+++	+
46	DSH	F	2y	Extranodal (skin)	+++	+++	+++
49	DSH	F	4Y	Extranodal (skin)	+++	+++	+++
54	DSH	M	5Y	Extranodal (nasal)	+++	+++	+++
71	DSH	F	5Y	Extranodal (nasal)	+++	+++	+++

DSH=Domestic short hair, M=Male, F=Female, Fs=Female sprayed, Mc=Male castrated, +++=Strongly positive, +=Mild positive, −=Negative

## Discussion

Previous studies have shown that B-cell lymphoma is more prevalent in canines [23], while T-cell lymphoma is more common in felines [24]. In the present study, the number of cats with B-cell lymphoma was more significant than that with T-cell lymphoma. In feline lymphomas, the proportional occurrence of subtypes differed from that observed in canine lymphomas. Specifically, while alimentary lymphoma was the most prevalent subtype in feline lymphomas [25], multicentric lymphoma was the most common form in canine lymphomas [26]. In contrast, this study found that most feline lymphoma cases were of the high-grade, multicentric B-cell lymphoma subtype. This differs from previous studies by Moore *et al*. [21] and Ii *et al*. [27], who suggested that alimentary lymphoma with T-cells was the most frequent neoplasm in feline lymphoma. Therefore, the subtype observed in this study was B-cell lymphoma.

IHC is not commonly used as the primary method for the definitive diagnosis of feline intestinal lymphoma in Thailand, possibly because some pet owners may not request it. Multicentric lymphoma is generally easier to diagnose in Thailand because it can collect cytology and biopsy samples from accessible sites. This study used more B-cell markers, CD20, CD79α, and PAX5, because the B-cell lineage confirmation did not involve the utilization of B-cell markers, including CD20(6). Previous studies by Mandara *et al*. [9] and Adams *et al*. [28] displayed routine analyses of paraffin-embedded tissues; an ideal B-cell lineage panel should include CD20 and CD79α and, optionally, PAX5 for mature lymphoid neoplasms.

Based on available data, extranodal lymphomas are the most diagnosed type of feline lymphoma and usually occur in old cats [29, 30]. Consequently, in this study, extranodal lymphoma emerged as the most diagnosed form of feline lymphoma. In addition, ocular and nasal lymphomas appear to be the most frequently observed subtype of extranodal lymphoma in cats. The most common immunophenotype of feline ocular lymphoma has B-cell origins [31]. This corroborates the findings of this study, which also reported that most feline ocular lymphoma cases were of the B-cell subtype, specifically the WHO classification of DLBCL [31].

Nasal lymphoma predominantly manifested in the nasal area rather than the nasopharyngeal region, which is consistent with a previous study by Santagostino *et al*. [32]. Our study also observed a predominance of small cell types with B-cell phenotype, aligned with previous investigations [33, 34]. Cases involving organs such as the spleen, liver, or nervous system usually categorized as extranodal lymphoma were not found in our study.

Renal lymphoma was found in 6% (5/86) of cases in this study, compared to an incidence of 3.6% (27/740) reported in a population of 740 cats diagnosed. Large B-cell lymphoma was predominantly observed, consistent with previous findings by Williams *et al*. [35].

In contrast to a previous study by Roccabianca *et al*. [36], where cutaneous lymphoma was predominantly of T-cell origin, our study observed both B-cell and T-cell phenotypes. In addition, this study did not find epitheliotropism, which is rare in cats. Our study did not find the criteria for cutaneous lymphoma at the injection site.

Multicentric lymphoma appears to be the second most common form of lymphoma in Thailand. In contrast, other studies have shown that the most common type of lymphoma is multicentric, predominantly affecting female cats [37]. Many previous studies [38, 39] have demonstrated that retrovirus infections are associated with feline mediastinal lymphoma. In a previous study by Versteegh *et al*. [40], it was found that mediastinal lymphoma associated with FeLV infection primarily involved T-cell lymphomas in young cats, while, B-cell lymphomas were associated with FIV infection [40]. Mediastinal and multicentric lymphomas are less common in countries where FeLV control and prevention measures are well-established [20, 37]. In our study, B-cell lymphoma was mainly observed in the mediastinal form of lymphoma; however, the potential role of rotaviruses in the development of mediastinal lymphoma was not discussed in this study.

A previous report by Moore *et al*. [21] found that alimentary lymphoma can be either B-cell or T-cell, depending on its location. In the previous study by Tidd *et al*. [41] and the current study, feline gastrointestinal lymphomas were explicitly classified as lymphoplasmacytic lymphomas and DLBCL. Regrettably, the present study did not accurately classify alimentary lymphoma subtypes, such as enteropathy-associated T-cell lymphoma Type I and enteropathy-associated T-cell lymphoma Type II [21, 42]. Histological analyses revealed the absence of mucosal epithelial cells, with observations limited to the submucosal and muscular layers of the small and large intestines. Due to constraints, a full-thickness biopsy was not feasible; however, a diagnosis was based on examining mesenteric lymph nodes and cytology, which exhibited multifocal enlargement alongside intestinal enlargement. In Thailand, diagnosing feline intestinal lymphoma can be challenging, as a full-thickness biopsy, an invasive diagnostic method, may not be feasible. Consequently, there are cases in which collecting a sample may not lead to a definitive diagnosis. A previous study by Felisberto *et al*. [14] demonstrated that approximately 84.6% of B-cells were positive for PAX5, and 100% were positive for CD79α. In contrast, the current study showed that 97% of B-cells were positive for PAX5. This discrepancy may be attributed to differences in the B-cell maturation stages of the various B-cell tumors examined, distinct sample processing methods utilized, and the potential for more aggressive biological behavior resulting from the loss of protein expression [14]. According to our study, PAX5 and CD3 markers are strongly recommended for diagnosing feline lymphoma. In cases where tissue samples exhibit cytoplasmic shrinkage, nuclear staining is crucial for assisting with the diagnostic process.

Histopathology with lymphoma classification, according to the WHO classification, is considered a fundamental method for diagnosing feline lymphoma [1]. This approach has proven to provide a significant survival advantage for cats, making it an essential tool in veterinary medicine.

Chino *et al*. [20] illustrated that T-cell alimentary lymphoma is prevalent in Japan, employing the updated Kiel classification rather than the WHO classification. Similarly, studies in Brazil [8] and the Netherlands [40] have highlighted alimentary lymphoma as the most common classification. In Europe, a previous study by Inazumi *et al*. [43] demonstrated the majority relevance of intestinal T-cell lymphoma. However, based on this study, the majority of cases intestinal lymphoma were B-cell lymphoma with DLBCL, similar to observations in Brazil [8]. This observation may stem from the sample’s location and this study’s inherent limitations.

DLBCL is the most prevalent subtype of feline lymphoma as observed by Leite-Filho *et al*. [8]. Significantly, relying solely on immunochemistry with one parameter may not be sufficient for a definitive diagnosis of B-cell lymphoma, as another parameter may also be necessary. Moreover, the immunophenotype of lymphoma can vary depending on its anatomical location and other factors, such as FeLV status and geographic region. The utilization of various B-cell markers in combination could aid pathologists in distinguishing between different stages of B-cell maturation, assessing tumor cell heterogeneity, and determining the phenotype in scenarios where there is a loss of common B-cell markers. The classification of feline lymphomas will prove valuable in predicting prognosis and developing tailored therapeutic protocols for each subtype of lymphoma.

## Conclusion

This study indicates that extranodal and multicentric lymphomas are the most frequent presentations found in cats in Bangkok. Mediastinal and alimentary lymphomas still occur. Nonetheless, the data for invasive collection samples are still inconclusive. In Thailand, cytology is the primary method for diagnosing lymphoma, with biopsies being employed to confirm inconclusive results. Biopsy procedures are typically conducted in specific areas, such as peripheral lymph nodes, skin, ocular sites, or nasal regions. This approach allows a more definitive diagnosis and aids in the appropriate management of the condition. The absence of data on intestinal lymphomas may contribute to its lack of identification as the most common subtype.

## Authors’ Contributions

JS: Investigation, methodology, validation, formal analysis, drafted and edited the manuscript SS and ST: Methodology, supervision, and writing, review, and editing of the manuscript. AR: Conceptualization, methodology, resources, supervision, writing, review, and editing of the manuscript. All authors have read, reviewed, and approved the final manuscript.
